# Academic Stress, Digital Strain, and Athlete Burnout Among Female Collegiate Basketball Players: A PLS-SEM and fsQCA Study

**DOI:** 10.3390/bs16101889

**Published:** 2026-10-13

**Authors:** Yuhang Lei, Wei Li, Li Cai

**Affiliations:** 1 School of Sports Science, Nanjing Normal University, Nanjing 210097, China; 231512039@njnu.edu.cn; 2 College of Physical Education, Huaqiao University, Quanzhou 362021, China; liwei0217@hqu.edu.cn

**Keywords:** athlete burnout, technostress, telepressure, partial least squares structural equation modeling (PLS-SEM), fuzzy-set qualitative comparative analysis (fsQCA)

## Abstract

Digital communication technologies expose student-athletes to implicit digital demands, yet their configurational impact alongside traditional stressors on burnout remains underexplored. Grounded in the JD-R model and Stress and Coping theory, this dual-method quantitative study (PLS-SEM and fsQCA) sampled female basketball players from the Chinese University Basketball Association League (CUBAL) to investigate how academic stress and digital strain associate with athlete burnout. PLS-SEM revealed that the composite construct of digital strain (integrating telepressure and technostress) acts as a significant indirect pathway linking academic stress and athlete burnout. Furthermore, fsQCA demonstrated that no single condition is necessary for high burnout; rather, specific combinations—such as high academic stress coupled with high techno-overload, or a purely “digital saturation” state—constitute configurations sufficiently associated with high burnout within this calibrated sample. Conversely, configurations associated with non-high burnout are asymmetrical, primarily relying on the concurrent absence of telepressure and techno-overload. Under the digital management paradigm, implicit digital strain acts as a critical proximal conduit for psychological exhaustion. The manifestation of high and non-high burnout is asymmetrical, depending on complex configurations of structural academic demands and digital expectations.

## 1. Introduction

Athlete burnout involves emotional and physical exhaustion and negatively relates to athletes’ well-being (Nicholls et al., 2022). The previous literature on athlete burnout has mainly focused on environmental stressors, such as physiological loads and competitive pressure (Edmonds et al., 2021). Recently, mobile communication technologies have changed sports team management. Offline synchronous management is shifting toward fragmented interactions based on instant messaging tools (Khetawat & Steele, 2023). Because many collegiate teams lack centralized physical training spaces, coaching staff rely on digital platforms for tactical deployment and member monitoring (Maiden et al., 2023). This keeps athletes in a continuous state of digital connectivity. However, empirical research has not fully clarified how digital management interacts with traditional stressors to associate with psychological strain.

This gap is evident among student-athletes, such as female basketball players in Division II of the Chinese University Basketball Association League (CUBAL) (Condello et al., 2019). These individuals hold dual identities as full-time undergraduate students and competitive athletes. Managing these concurrent roles creates conflicts in the allocation of limited time and cognitive energy (Yukhymenko-Lescroart, 2014). To capture the multiple pressures faced by this population, this study uses a cross-sectional, dual-method quantitative approach (PLS-SEM and fsQCA). By integrating variance-based and configurational analyses, this study investigates how academic stress and specific dimensions of digital strain concurrently associate with athlete burnout.

This section delineates the theoretical foundation of the study. It first explores the dual identity of student-athletes and conceptualizes academic stress within the Job Demands–Resources (JD-R) model. Subsequently, it examines the transition of digital communication from a resource to a demand, conceptualizing “Digital Strain,” and outlines the dual-method approach used to form the research hypotheses.

### 1.1. Theoretical Foundation and Research Framework

#### 1.1.1. Dual Identity and Academic Stress in the JD-R Model

The Job Demands–Resources (JD-R) model categorizes organizational environments into job demands and job resources, where sustained demands continuously deplete physiological and psychological resources (Bakker & Demerouti, 2017). For student-athletes, academic stress should be conceptualized as a structural “job demand” rather than a transient educational challenge (De Brandt et al., 2025). Student-athletes must meet standardized academic assessment requirements. Institutional rules require them to maintain specific GPA thresholds to keep their athletic eligibility. Balancing these academic loads with rigorous daily training tasks is a mandatory structural constraint. This cross-domain pressure chronically drains athletes’ cognitive and emotional resources, predisposing them to exhaustion (Sorkkila et al., 2017).

#### 1.1.2. The Conceptualization of Digital Strain: Merging Telepressure and Technostress

In the digitalized sports environment, digital communication has a bivalent nature. Initially, digital tools can act as resources by facilitating coordination and tactical sharing (Gurgis & Kerr, 2021). However, the power asymmetry between coaches and student-athletes can transform team notifications into psychological demands. The expectation to be “always on” blurs the boundaries between the athletic role, the academic role, and personal recovery time (Tarafdar et al., 2020).

The previous literature often assesses the burdens of digital connectivity via two distinct constructs: telepressure (the internal urge to respond) and technostress (the external perception of technology-induced overload and conflict) (Ayyagari et al., 2011; Barber & Santuzzi, 2015). However, in this context, these two phenomena demonstrate extreme conceptual and empirical overlap. Treating them as independent variables violates the assumption of discriminant validity and artificially inflates multicollinearity. Based on the Stressor–Strain–Outcome (SSO) framework (Kahn & Byosiere, 1992), which focuses on the transition from environmental stimuli to psychological strain, this study collapses these elements into a single, higher-order composite construct termed “Digital Strain.” This construct represents the shared variance of the digital burden, integrating the internal urge to respond and the external cognitive overload. This resolves multicollinearity while maintaining the underlying theoretical structure.

### 1.2. Hypotheses Development

#### 1.2.1. PLS-SEM Hypotheses (Net Mediating Effects)

Based on the resource depletion mechanism of the JD-R model (Bakker & Demerouti, 2017), academic stress constitutes a primary structural job demand for student-athletes. The mandatory requirement to maintain academic eligibility continuously consumes their limited physiological and cognitive resources, directly predisposing them to elevated risks of exhaustion.

**Hypothesis** **1 (H1).** *Academic stress is positively associated with athlete burnout.*

Furthermore, this structural pressure exhibits a cross-domain spillover effect, increasing the baseline psychological load when student-athletes process digital team tasks (Sorkkila et al., 2017). Consequently, higher academic demands are expected to exacerbate the perceived intensity of digital strain.

**Hypothesis** **2 (H2).** *Academic stress is positively associated with digital strain.*

**Figure 1:**
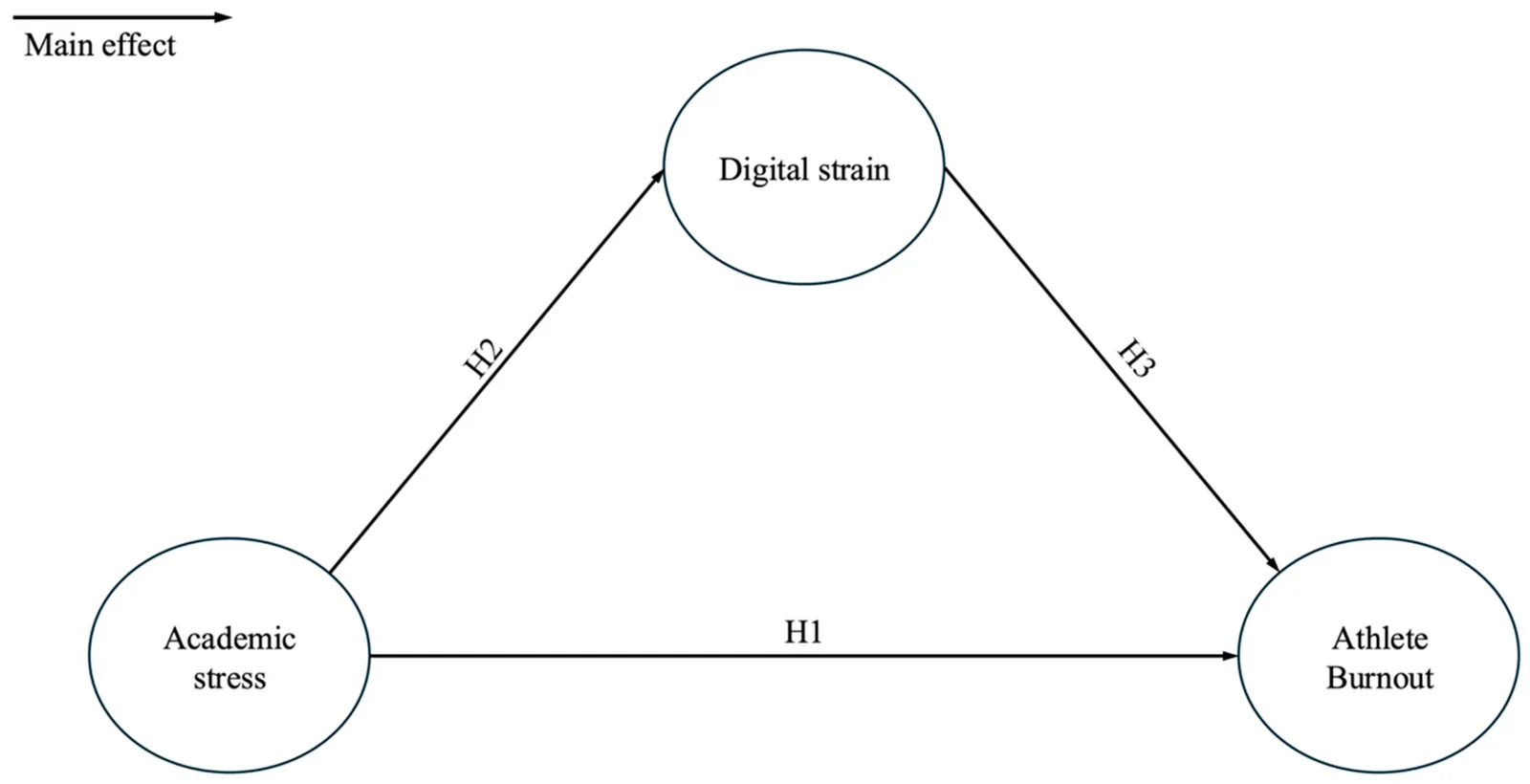
Solid arrows indicate linear predictive paths. The mediating role of digital strain hypothesized in H4 is embedded within the direct pathways (H1, H2, H3). The multiple concurrent and asymmetrical configurational associations explored by the subsequent fsQCA in this study are not depicted in this linear model diagram.

Within the SSO framework (Kahn & Byosiere, 1992), sustained digital strain—representing the internalized pressure to remain connected and the subsequent cognitive overload—functions as an acute psychological strain. Without adequate recovery, this continuous cognitive overextension substantively relates to the manifestation of chronic emotional and physical exhaustion (Tarafdar et al., 2020) (Figure 1).

**Hypothesis** **3 (H3).** *Digital strain is positively associated with athlete burnout.*

Integrating the aforementioned pathways, while academic stress provides the structural basis for resource depletion, it necessitates the internalization of digital strain to substantially translate into chronic exhaustion under the digital management paradigm. Therefore, digital strain serves as the essential mediating mechanism linking tangible academic demands to psychological burnout.

**Hypothesis** **4 (H4).** *Digital strain mediates the positive association between academic stress and athlete burnout.*

#### 1.2.2. fsQCA Research Propositions

While PLS-SEM is optimal for capturing net mediating effects, traditional linear models overlook the diverse combinatorial patterns and asymmetrical nature of environmental variables (Pappas & Woodside, 2021). Burnout among student-athletes is rarely driven by a single factor; it is the product of concurrent interactions (Misangyi et al., 2016). To address this, we use fsQCA to disaggregate the “Digital Strain” composite into its specific sub-dimensions (e.g., telepressure, techno-overload, and techno-conflict) to explore their configurational associations with academic stress. Given the cross-sectional data, the focus is on configurational associations rather than temporal causality.

**Proposition** **1.** *No single condition is necessary to associate with high athlete burnout; rather, multiple alternative configurations of academic stress and the distinct sub-dimensions of digital strain are sufficient to associate with high athlete burnout.*

**Proposition** **2.** *The configurational associations for high athlete burnout and non-high athlete burnout exhibit configurational asymmetry, indicating that the configurations associated with non-high burnout are not simple inverses of the configurations associated with high burnout.*

## 2. Materials and Methods

This section outlines the empirical research design and methodological procedures of the study. It first describes the adaptation and validation of the measurement instruments. Subsequently, it details the participant characteristics, the sampling strategy, and the data collection process. Finally, it presents the data analysis strategy, elaborating on the dual-method quantitative approach (PLS-SEM and fsQCA).

### 2.1. Measurement Instruments

All measurement instruments utilized in this study were adapted from established scales in the extant literature. To ensure semantic equivalence and contextual applicability for CUBAL female basketball players, a standard translation-back-translation procedure was executed. Subsequently, cognitive interviews utilizing a think-aloud protocol were conducted with target participants to refine item phrasing and eliminate contextual ambiguities. Concurrently, a panel of three domain experts evaluated the retained items to confirm content validity (Beatty & Willis, 2007). To mitigate potential common method bias during data collection, items sharing identical Likert-scale anchors were randomized in the questionnaire design.

Academic stress: Academic stress was assessed using an adapted version of the Perception of Academic Stress (PAS) scale (Bedewy & Gabriel, 2015). To adhere to the principle of model parsimony and focus on the structural demands specific to student-athletes, the original scale was systematically shortened. Specifically, items assessing general future career anxiety were removed, as they lack relevance to the immediate structural constraints of athletic eligibility. Only four core items explicitly reflecting dual-role time constraints and academic workload were retained (e.g., an item originally concerning ‘exams and deadlines’ was adapted to reflect ‘balancing exam preparation with training schedules’; an item on ‘time pressure from coursework’ was retained to capture the structural demand of maintaining athletic eligibility).

Digital Strain (Composite Mediator): Consistent with the Stressor–Strain–Outcome (SSO) framework (Kahn & Byosiere, 1992) and to effectively capture the holistic digital burden, telepressure (the internal urge to respond) and technostress (the external perception of overload and conflict) were conceptualized as overlapping dimensions of a single higher-order composite construct termed ‘Digital Strain’.

The telepressure dimension was measured using the workplace telepressure scale (Barber & Santuzzi, 2015), capturing the internal compulsion to respond immediately to asynchronous team notifications.

The technostress dimension was adapted from the technostressors scale (Tarafdar et al., 2007). Based on cognitive interviews and the specific context of utilizing ubiquitous instant messaging applications (e.g., WeChat), only the techno-overload and techno-conflict dimensions were retained. The techno-complexity and techno-insecurity dimensions were omitted because they primarily apply to complex enterprise software rather than highly familiar mobile applications. Crucially, while the classical techno-invasion dimension (reflecting an “always-on” culture) is vital, its conceptual and empirical domain in this study was directly captured by the telepressure scale, which assesses the psychological invasion of boundaries. Theoretically, merging these dimensions into a unified ‘Digital Strain’ construct is justified because telepressure captures the internal psychological urge to remain responsive, whereas technostress (overload and conflict) reflects the external role demands; together, they function as a holistic stressor that depletes athletes’ cognitive resources, moving beyond mere statistical necessity. All items were assessed using a 5-point Likert scale.

Athlete burnout: Athlete burnout was measured utilizing the Athlete Burnout Questionnaire (ABQ) (Raedeke & Smith, 2001). The complete 15-item scale, encompassing three dimensions (reduced sense of accomplishment, devaluation, and emotional/physical exhaustion), was retained to ensure standardized measurement. Items were evaluated on a 5-point Likert scale. The complete list of retained measurement items is provided in the Supplementary Materials.

### 2.2. Participants and Data Collection

Participants were recruited from female collegiate basketball teams competing in Division II of the Chinese University Basketball Association League (CUBAL) within a specific province. Given the stringent inclusion criteria—active roster status, female gender, and full-time undergraduate enrollment—the overall target population in this specific competitive tier is inherently highly bounded. A total of 80 questionnaires were distributed, and 67 valid responses were retained after excluding incomplete submissions (yielding an effective response rate of 83.75%). While the final sample size (N = 67) is undeniably small, it is mathematically sufficient for the rigorously streamlined models employed in this revised study. First, to ensure model parsimony and preserve statistical power within the constrained sample size (N = 67), highly correlated digital stress variables were collapsed into a single composite mediator (Digital Strain). Regarding sample size justification, a sensitivity power analysis utilizing the inverse square root method (Kock & Hadaya, 2018) was conducted. Given the final sample size (N = 67), the analysis demonstrates that the current sample provides 80% statistical power at a 5% significance level to reliably detect a minimum absolute path coefficient of 0.304. Consequently, while the sample is adequately powered to detect the weakest theoretically important direct effect within the model (Digital Strain → Athlete Burnout, β = 0.347), it lacks the conventional 80% statistical power required to definitively establish smaller indirect associations (e.g., the mediation path, β = 0.245). Acknowledging these specific statistical power constraints and the cross-sectional design, this structural analysis is strictly framed as an exploratory investigation. Second, regarding the configurational analysis, fsQCA is methodologically designed to handle small-to-medium-N scenarios (typically ranging from 15 to 50 cases), rendering a sample of 67 highly appropriate for set-theoretic exploration (Pappas & Woodside, 2021). Nevertheless, we explicitly acknowledge that with N = 67, path estimates, bootstrap confidence intervals, and fsQCA configurations remain sensitive to individual case variations. Consequently, rather than determining universal causal laws, the findings drawn from this sample are conservatively interpreted as specific, context-bound configurational associations that require future replication in larger cohorts. Data collection was conducted in May 2024. Prior to questionnaire administration, written informed consent was obtained from all participants and the respective coaching staff. The survey was administered anonymously to minimize social desirability bias, and athletes were explicitly assured that their responses would not affect their team standing. Demographic data—including age, training tenure, and academic major—were collected. Crucially, to proactively prevent model specification errors, these demographic characteristics were comprehensively incorporated as control variables in the subsequent PLS-SEM structural model, regardless of their univariate non-significance with the outcome variables.

Demographic characteristics of the sample are detailed in Table 1. The sample encompassed various academic grades, ranging from undergraduate to postgraduate levels, with lower-grade players (freshmen and sophomores) accounting for 56.7%. Regarding athletic classification, this study categorized the respondents into two groups based on whether they had obtained official certification from national sports administrative departments: “unclassified” and “classified” (possessing official athletic ranks). For the playing tenure variable, a categorical segmentation approach was employed to categorize the original continuous data, operationalizing it into three groups: short, medium, and long tenure. Additionally, statistical data revealed that approximately 43% of the athletes spent more than 30 min daily managing digital notifications from the team, which reflects the high level of digital connectivity demands faced by the target population.

### 2.3. Data Analysis Strategy

Given the objective of exploring both net mediating effects and specific configurational patterns, this study adopted a dual-method quantitative strategy. The analytical procedures were executed in three sequential phases. First, IBM SPSS Statistics (Version 27.0, IBM Corp., Armonk, NY, USA) was utilized for data screening, descriptive statistics, and the assessment of common method bias. Crucially, this phase also included the computation of raw mean scores for each variable, derived directly from the corresponding items of the measurement scales, to serve as the foundational data for subsequent set-theoretic analysis. Second, Partial Least Squares Structural Equation Modeling (PLS-SEM) was conducted using SmartPLS 4.0 (SmartPLS GmbH, Oststeinbek, Germany). PLS-SEM was selected because it maximizes the explained variance of endogenous constructs and demonstrates robust statistical performance when handling relatively small sample sizes and complex composite models (Hair et al., 2019). This variance-based approach was utilized strictly to examine the linear mediating pathways linking academic stress, the composite variable of digital strain, and athlete burnout, while statistically controlling for demographic variables. Third, Fuzzy-set Qualitative Comparative Analysis (fsQCA) was performed using fsQCA 3.0 software (University of California, Irvine, CA, USA). To strictly prevent circular validation and methodological confounding between the variance-based and set-theoretic analyses, the fsQCA calibration procedure exclusively utilized the raw mean scores calculated directly from the survey items, deliberately discarding the latent variable scores derived from the PLS-SEM estimation (Pappas & Woodside, 2021). fsQCA was applied to identify sufficient configurations of academic and digital demands associated with high burnout. To address potential data instability associated with the constrained sample size (N = 67), the complementary use of fsQCA is not intended to establish universal deterministic rules; rather, the results are interpreted cautiously as context-specific asymmetrical patterns.

## 3. Results

This section presents the empirical findings of the study. It begins with preliminary data screening and descriptive statistics, followed by the systematic evaluation of the measurement and structural models via PLS-SEM, and concludes with the configurational analysis utilizing fsQCA.

### 3.1. Common Method Bias Testing and Descriptive Statistics

Given that all data were self-reported via a cross-sectional survey, Harman’s single-factor test was conducted in SPSS 27.0 to assess the potential threat of Common Method Bias (CMB). An unrotated principal component analysis encompassing all retained items for academic stress, digital strain, and athlete burnout revealed that the first single factor accounted for 41.11% of the total variance. As this value falls comprehensively below the conservatively recommended threshold of 50% (Podsakoff et al., 2003), CMB does not constitute a pervasive issue in this dataset.

Methodologically, to maintain conceptual clarity across different analytical techniques, Digital Strain was strictly operationalized as a higher-order construct. Specifically, within the PLS-SEM structural analysis, it was estimated utilizing the disjoint two-stage approach (Sarstedt et al., 2019). Conversely, for the preliminary bivariate correlations and the subsequent fsQCA calibration procedure, it was operationalized via the mathematical average of the items retained strictly after the PLS-SEM purification. Utilizing this purified item set ensures measurement equivalence between the variance-based and set-theoretic analyses, deliberately excluding noise indicators with established low reliability. Table 2 presents the means, standard deviations, and Pearson correlation coefficients for all core theoretical constructs.

The bivariate correlation analysis indicates that academic stress is significantly and positively correlated with digital strain (r = 0.697, *p* < 0.01) and athlete burnout (r = 0.393, *p* < 0.01). Furthermore, digital strain exhibits a significant positive correlation with athlete burnout (r = 0.472, *p* < 0.01). Notably, training tenure showed a significant negative correlation with athlete burnout (r = −0.255, *p* < 0.05). Additionally, one-way ANOVA analyses revealed statistically significant differences in digital strain across varying grade levels (*p* = 0.048) and sports qualification levels (*p* = 0.014). Subsequent Least Significant Difference (LSD) post hoc comparisons indicated that juniors (Grade 3) reported significantly higher digital strain than both freshmen (Grade 1, *p* = 0.004) and graduate students (Grade 5, *p* = 0.050). Post hoc analyses for sports qualification levels were precluded due to restricted subgroup sample sizes (e.g., n = 1 for the elite category). No significant differences were observed for athlete burnout across these categorical groups (all *p* > 0.05).

Following best practices for proper model specification (Bernerth & Aguinis, 2016), demographic factors were included as control variables based on their theoretical potential to confound the structural relationships, independent of their bivariate statistical significance. Prior research establishes that extended training tenure often corresponds with cumulative physical and emotional depletion, inherently influencing baseline burnout levels. Similarly, grade level dictates the intensity of academic demands, which structurally alters the athlete’s perception of digital strain. Furthermore, the daily duration spent handling team notifications serves as an objective proxy for the total volume of digital exposure. Empirical evidence consistently demonstrates that prolonged temporal engagement with digital demands systematically increases cognitive load, thereby directly exacerbating the subjective perception of digital strain (Khetawat & Steele, 2023; Tarafdar et al., 2020). Adhering to parsimonious control practices in small-sample structural equation modeling (Becker et al., 2016), training tenure and daily team communication time were strictly incorporated as continuous variables, while grade level was processed via dummy coding prior to its inclusion in the multivariate structural model.

### 3.2. Measurement Model Assessment

To appropriately evaluate the higher-order constructs (HOCs) of digital strain and athlete burnout, the disjoint two-stage approach was employed in SmartPLS 4 (Sarstedt et al., 2019). This procedure necessitates a systematic evaluation of the measurement model at both the first-order (lower-order components) and second-order (higher-order components) levels.

#### 3.2.1. Reliability and Convergent Validity Assessment of the Measurement Model

First, indicator reliability was examined. To ensure measurement precision, an item purification procedure was conducted wherein four specific indicators with outer loadings below 0.60 were explicitly eliminated from the model. Specifically, the removed indicators comprised two items from the athlete burnout scale (e.g., ‘I am accomplishing many tasks as a basketball player, but I do not care if I am successful’, ‘I feel I have achieved many valuable things in basketball’), one item from the technostress scale (‘The team’s digital communication demands force me to process tasks at a faster pace’), and one item from the telepressure scale (‘I often think about needing to respond to team messages promptly’). Following this refinement, the retained first-order indicators exhibited robust outer loadings ranging from 0.661 to 0.911. Specifically, item ab7 (‘I feel I am not realizing my full potential in basketball’; loading = 0.661) was retained, as Hair et al. (2019) stipulate that indicators with loadings between 0.40 and 0.708 must not be deleted if their removal compromises the construct’s internal consistency reliability. Analytical testing confirmed that deleting this item (ab7) would precipitate a critical drop in the Cronbach’s α of its respective dimension to 0.68, violating the fundamental α > 0.70 criterion. Consequently, this item was retained to preserve both the theoretical content validity and the statistical structural integrity of the dimension. At the second-order level, the outer loadings of the dimensions on their respective HOCs ranged from 0.733 to 0.942, demonstrating excellent indicator reliability (see Table 3).

Internal consistency reliability and convergent validity were subsequently evaluated. As presented in Table 3, at the first-order level, Cronbach’s α values ranged from 0.774 to 0.889, Composite Reliability (CR, ρc) from 0.843 to 0.919, and Average Variance Extracted (AVE) from 0.646 to 0.720. At the second-order level, the Cronbach’s α for all core constructs ranged from 0.831 to 0.908, CR values ranged from 0.897 to 0.942, and AVE values ranged from 0.719 to 0.844. All metrics comprehensively exceeded the stringent thresholds of 0.70 for reliability and 0.50 for AVE (Hair et al., 2019), thereby confirming robust internal consistency and convergent validity across both measurement levels.

#### 3.2.2. Discriminant Validity Assessment of the Measurement Model

Discriminant validity was established using the Heterotrait–Monotrait ratio of correlations (HTMT) criterion (Henseler et al., 2015). During the initial evaluation at the first-order level, specific dimensions exhibited HTMT values exceeding the conservative 0.85 threshold (e.g., HTMT for Techno-overload and Techno-conflict = 0.928; HTMT for Emotional Exhaustion and Devaluation = 0.923). This empirical evidence of substantial conceptual overlap provided direct methodological justification for abstracting these specific dimensions into the respective higher-order constructs of Digital Strain and Athlete Burnout.

Following the application of the disjoint two-stage approach, discriminant validity was reassessed at the structural model level. As illustrated in Table 4, the highest HTMT value among the final structural constructs was 0.793 (between Academic Stress and Digital Strain). Given that all HTMT values fell strictly below the conservative threshold of 0.85, discriminant validity among the core variables is robustly established.

Furthermore, to address potential multicollinearity concerns, the Variance Inflation Factor (VIF) was examined for all predictor constructs within the revised structural model. The VIF values for the structural paths ranged from 1.484 to 3.180. Falling comprehensively below the conservative threshold of 3.3 (Diamantopoulos & Siguaw, 2006), these results robustly rule out severe collinearity interference in the estimation of the structural paths.

### 3.3. Structural Model Path Hypothesis Testing

To evaluate the structural model and test the hypothesized relationships, a bootstrapping procedure with 5000 subsamples was executed using SmartPLS 4 (Hair et al., 2019). Given the cross-sectional nature of the data, the structural analyses test for statistical associations and predictive relationships rather than absolute causality.

To address potential confounding effects and ensure model robustness (Spector & Brannick, 2011), the structural model was evaluated in two stages: a base model (excluding control variables) and a controlled model incorporating relevant demographic variables (training tenure, team-related communication time, and grade). Adhering to parsimonious control practices in small-sample structural equation modeling (Becker et al., 2016), the coding schemes for the control variables were strictly specified to conserve degrees of freedom while maintaining analytical accuracy. Specifically, training tenure (years) and team-related communication time (hours per day) were incorporated as continuous variables. Conversely, grade level was processed via dummy coding prior to its inclusion in the structural model. Categorical variables exhibiting extreme class imbalance (i.e., sports level, with the specified category comprising only 8.9% of the sample) were excluded to prevent zero-variance issues and singular matrix errors during the bootstrapping algorithm calculation (Tabachnick & Fidell, 2013). The structural estimation results for both models are presented in Table 4.

The explanatory power of the fully controlled structural model was assessed first. The model accounted for 55.0% of the variance in digital strain (R^2^ = 0.550) and 34.2% of the variance in athlete burnout (R^2^ = 0.342), indicating substantial and moderate predictive accuracy, respectively (Hair et al., 2019). Furthermore, the corresponding effect sizes (f^2^) for the primary predictive paths (Academic Stress → Digital Strain: f^2^ = 1.072; Digital Strain → Athlete Burnout: f^2^ = 0.082) surpassed the recommended thresholds for large and small effects.

Subsequently, the direct and indirect structural paths were evaluated. In the controlled model, the results demonstrated a significant positive association between academic stress and digital strain (β = 0.706, t = 9.965, *p* < 0.001, 95% CI [0.556, 0.835]), supporting Hypothesis 2. Digital strain, in turn, was significantly and positively related to athlete burnout (β = 0.347, t = 2.104, *p* = 0.035, 95% CI [0.013, 0.668]), supporting Hypothesis 3. Conversely, the direct path coefficient from academic stress to athlete burnout was statistically non-significant (β = 0.164, t = 1.048, *p* = 0.295, 95% CI [−0.129, 0.484]), thus rejecting Hypothesis 1.

**Figure 2:**
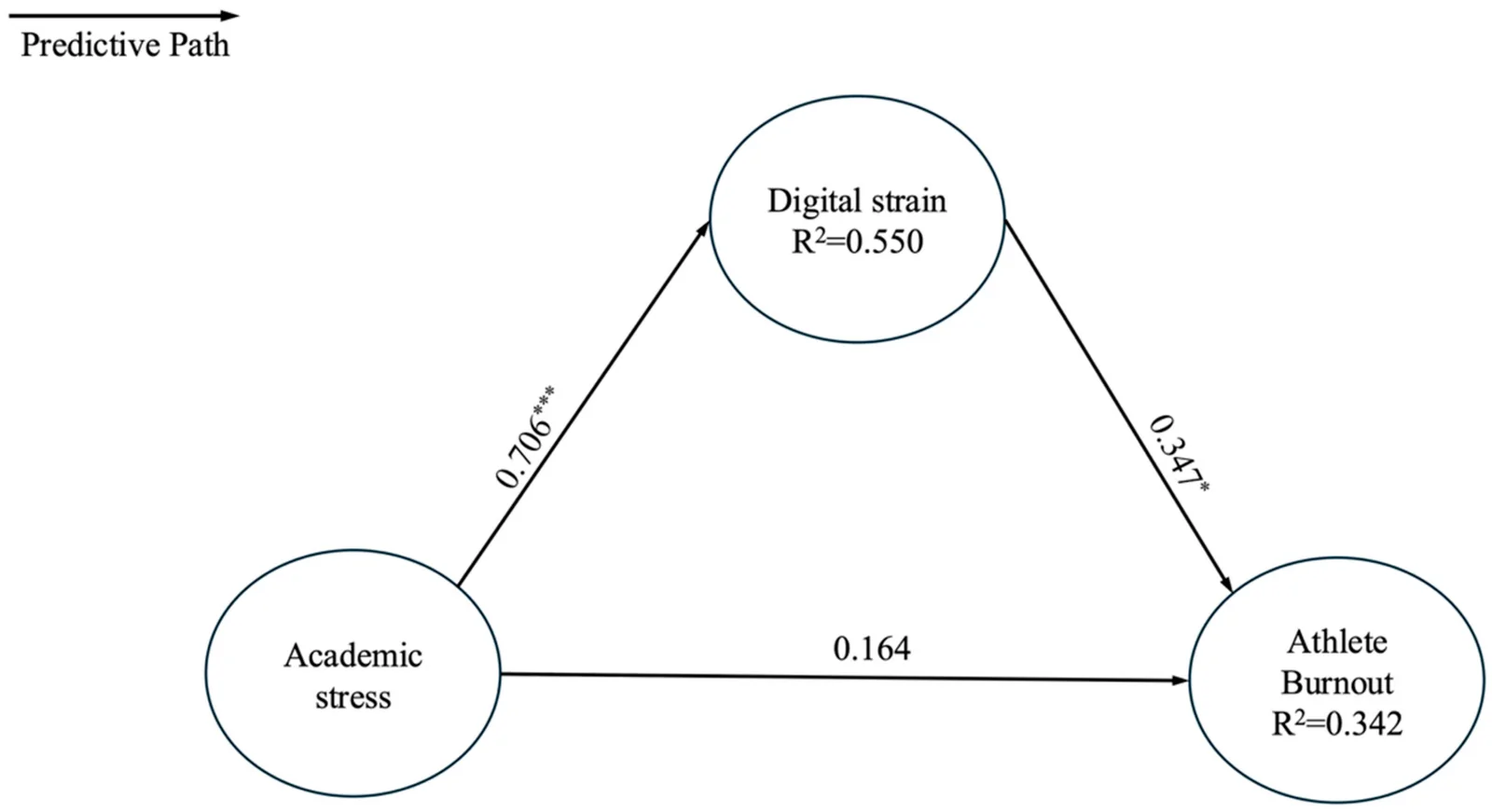
Structural model results and path coefficients. Note: AB = Athlete burnout; AS = Academic stress; DS = Digital strain. β values are standardized path coefficients. R^2^ values indicate the proportion of variance explained in the endogenous constructs. * *p* < 0.05, *** *p* < 0.001.

The structural analysis indicated that digital strain constitutes a significant indirect pathway in the relationship between academic stress and athlete burnout (indirect effect β = 0.245, t = 2.102, *p* = 0.036, 95% CI [0.01, 0.473]). According to the mediation typology established by Zhao et al. (2010), the presence of a significant indirect path alongside a non-significant direct path characterizes an indirect-only pattern in this sample. This structural configuration was achieved through unconstrained empirical estimation without artificially fixing any paths to zero, thereby structurally supporting Hypothesis 4. As illustrated in Table 5 and Table 6, the magnitude and significance of these core paths remained consistent across both the base and controlled models, verifying that the hypothesized relationships are independent of the specified demographic variations (Figure 2).

### 3.4. Fuzzy-Set Qualitative Comparative Analysis (fsQCA)

This study employed fsQCA 3.0 software to conduct configurational analysis on the data, aiming to explore the complex configurational patterns by which academic stress and specific digital demands associate with high and non-high athlete burnout.

#### 3.4.1. Data Calibration and Theoretical Direction

To address the potential masking of complex interactions within aggregated constructs and achieve genuine methodological triangulation, a fuzzy-set qualitative comparative analysis (fsQCA) was conducted. To avoid merely repackaging the PLS-SEM net-effect findings, the aggregated “digital strain” construct was theoretically disaggregated into specific digital demands: telepressure, techno-overload, and techno-conflict (Tarafdar et al., 2007). This disaggregation allows for the examination of how distinct digital demands configure with academic stress to form sufficient conditions associated with athlete burnout.

Strictly adhering to fsQCA protocols, the calibration process utilized the mathematical average of the specific scale items retained exclusively after the PLS-SEM purification process for each condition. This approach prevents the reintroduction of low-loading items into the fsQCA, thereby maintaining strict measurement equivalence across both analytical stages, while deliberately avoiding latent variable scores derived from the structural model (Greckhamer et al., 2018). Given the absence of absolute clinical cut-offs for academic and digital stress in student-athletes, the continuous variables were calibrated into fuzzy-set membership scores using the sample’s empirical percentiles: 95th (full membership), 50th (crossover point), and 5th (full non-membership). To ensure absolute methodological transparency, the actual calibration anchors (95th, 50th, and 5th) were strictly defined as follows: Athlete Burnout (3.308, 2.501, and 1.333), Academic Stress (4.675, 3.251, and 1.325), Telepressure (5.000, 3.601, and 1.660), Techno-overload (4.750, 3.001, and 1.500), and Techno-conflict (4.333, 3.334, and 1.100). This data-driven calibration is standard practice in behavioral sciences to capture the relative intensity of stressors within a specific population (Ragin, 2009).

#### 3.4.2. Necessity Analysis

Prior to constructing the truth table, the necessity of individual conditions for high athlete burnout (FZAB) was assessed. According to Ragin (2009), a condition is deemed necessary if its consistency exceeds the 0.90 threshold. The analysis revealed that the highest consistency value was 0.796 (techno-overload), followed by academic stress (0.772) and techno-conflict (0.778). Consequently, no single condition constitutes a necessary prerequisite for high athlete burnout, warranting the subsequent sufficiency analysis of condition configurations. The full truth table utilized for this analysis is detailed in Appendix A.

#### 3.4.3. Sufficiency Configuration and Configurational Asymmetry Analysis

The sufficiency analysis for high athlete burnout (FZAB) and the absence of high athlete burnout (~FZAB) was conducted using a frequency threshold of 1. During truth table minimization to obtain the intermediate solution, directional expectations (counterfactual analysis) were applied based on the Job Demands–Resources (JD-R) model. Specifically, it was assumed that the presence of demands (academic stress, telepressure, techno-overload, and techno-conflict) contributes to burnout, while their absence contributes to the absence of burnout.

To ensure rigorous sufficiency, the raw consistency threshold for FZAB was set to > 0.82 (with PRI > 0.50). For the negated outcome (~FZAB), the research faced a methodological trade-off regarding the consistency cut-off. While raising the threshold could mathematically artificially inflate the solution consistency above 0.80, doing so would necessitate excluding a critical truth table row containing a substantial cluster of empirical cases (N = 12). To explicitly prevent configurations from being driven by small, isolated cases—a significant risk in samples of this size—this core cluster was consciously retained. This conservative adjustment yielded an exploratory solution with substantial case representation and a strictly acceptable overall consistency of 0.792 (well above the 0.75 minimum recommended by Ragin, 2009).

Two configurations (H1, H2) were identified as sufficiently associated with high burnout within this calibrated sample, yielding a solution coverage of 0.757 and a solution consistency of 0.822.

Configuration H1 (fzas * fzdsoverload) indicates that the presence of high academic stress and high techno-overload is sufficiently associated with high burnout within this calibrated sample (Consistency = 0.824, Raw Coverage = 0.686).

Configuration H2 (fzdstele * fzdsoverload * fzdsconflict) demonstrates that the simultaneous presence of high telepressure, high techno-overload, and high techno-conflict constitutes an alternative configuration sufficiently associated with high burnout within this calibrated sample (Consistency = 0.855 and Raw Coverage = 0.667).

In accordance with configurational asymmetry, the conditions associated with the absence of burnout are not simply the mirror opposites of those associated with its presence. The analysis of ~FZAB revealed two distinct configurations (L1 and L2) with a solution consistency of 0.792 and a high solution coverage of 0.808.

Configuration L1 (~fzdsoverload) constitutes a primary mitigating configuration, indicating that the singular absence of techno-overload is broadly associated with the absence of high burnout across diverse contexts (Raw Coverage = 0.783).

Configuration L2 (~fzas * ~fzdstele * ~fzdsconflict) demonstrates that even when techno-overload is present, the concurrent absence of academic stress, telepressure, and techno-conflict serves as an alternative highly consistent configuration (Consistency = 0.859) associated with non-high burnout (Table 7).

#### 3.4.4. Robustness Checks

To verify the stability of the truth table and ensure the findings are not artifacts of the chosen parameters, sensitivity analyses were performed by adjusting the calibration anchors from the 95th, 50th, and 5th percentiles to the 90th, 50th, and 10th percentiles (Greckhamer et al., 2018). For the positive outcome (FZAB), the robustness check yielded identical core configurations (AS * Overload; Telepressure * Overload * Conflict) with a stable solution consistency of 0.814. For the negated outcome (~FZAB), the adjusted thresholds produced three configurations (solution consistency = 0.804) that consistently emphasized the absence of telepressure and techno-overload as fundamental to avoiding burnout. The substantive interpretation of the alternative configurational pathways remained entirely consistent across different calibration metrics, confirming the robust validity of the configurational models. Furthermore, an additional sensitivity fsQCA conducted using the mathematical averages of the original, unpurified scale items yielded the exact same core configurational pathways for high athlete burnout (i.e., configurations H1 and H2; solution consistency = 0.815, solution coverage = 0.757), further strengthening confidence in the results.

## 4. Discussion

This section contextualizes the empirical findings within the extant theoretical literature. It sequentially discusses the linear structural relationships and the specific configurational pathways, followed by an articulation of the theoretical contributions and practical implications for collegiate sports management.

### 4.1. Discussion of Results

#### 4.1.1. Structural Relationships and the Indirect Role of Digital Strain

The variance-based PLS-SEM analysis provides initial empirical evidence elucidating how the dual-role demands of student-athletes associate with psychological exhaustion within a digitalized management context. Consistent with the Job Demands–Resources (JD-R) model, the findings indicate that academic stress, representing a structural job demand, is significantly and positively associated with digital strain (H2). This aligns with recent scholarly assertions that the cognitive load imposed by academic obligations significantly depletes student-athletes’ baseline psychological resources (Sorkkila et al., 2017). When athletes operate under continuous academic pressure, their threshold for tolerating additional cognitive loads—such as the expectation to process fragmented digital team instructions—is markedly lowered, thereby exacerbating the perceived intensity of digital strain (De Brandt et al., 2025).

Furthermore, the results support the positive association between digital strain and athlete burnout (H3). While previous literature has predominantly focused on physical overtraining or traditional interpersonal conflicts (Edmonds et al., 2021), the current findings highlight digital strain as a critical contemporary stressor. By integrating telepressure and technostress into a composite construct, this study captures the specific reality of modern collegiate sports teams. Because collegiate programs often lack centralized physical training facilities, coaching staff increasingly rely on digitalized tactical deployments and constant monitoring (Maiden et al., 2023; Khetawat & Steele, 2023). The internalized urge to respond immediately, coupled with the cognitive overload of processing these tasks outside of regular training hours, acts as an acute strain that significantly associates with emotional and physical exhaustion (Tarafdar et al., 2020; Nicholls et al., 2022).

Regarding the indirect effect (H4), the model demonstrates that digital strain serves as a significant conduit linking academic stress to athlete burnout. However, adhering to rigorous methodological interpretations, these results must be viewed with caution. Given the cross-sectional design of the study and the necessary model specifications adopted to resolve multicollinearity, the findings are consistent with a robust indirect role of digital strain but do not provide definitive evidence of absolute mediation. Instead, the data are consistent with an indirect-only pattern in this sample, suggesting that while chronic academic demands provide the structural foundation for resource depletion, the boundary-blurring nature of digital team demands acts as the primary proximal mechanism through which this depletion translates into observable exhaustion.

#### 4.1.2. Configurational Pathways Associated with Athlete Burnout

To capture the complex interactions of these stressors, the fsQCA identified sufficient condition configurations that co-occur with athlete burnout. Notably, this is because the calibration of these configurations relies on the internal percentiles of this specific sample (N = 67). Therefore, the findings reflect relative stress intensities within this collegiate cohort and represent exploratory mechanisms requiring replication in larger populations, rather than absolute clinical thresholds.

The analysis identified two empirical pathways associated with high athlete burnout (H1 and H2). Configuration H1 (High Academic Stress * High Techno-overload) highlights the acute time-resource conflict inherent in the student-athlete dual identity. When the structural demands of academic obligations consume substantial cognitive resources, athletes possess a diminished buffering capacity. The addition of high techno-overload creates a resource deficit that is sufficiently associated with the co-occurrence of high burnout (Stambulova et al., 2021; Edú-Valsania et al., 2022). Configuration H2 (High Telepressure * High Techno-overload * High Techno-conflict) illustrates a purely “digital saturation” pathway. This suggests that when athletes internalize a strict expectation to remain constantly online, are exposed to a high volume of digital demands, and experience digital intrusions into recovery time, emotional exhaustion occurs even without salient academic stress. This supports recent arguments that ubiquitous digital connectivity in sports can function as a standalone severe stressor (Khetawat & Steele, 2023).

Consistent with configurational asymmetry, the conditions mitigating burnout are not merely the mirror opposites of those associated with it. The analysis of the absence of high burnout (~FZAB) yielded two distinct mitigating configurations (L1 and L2). Notably, Configuration L1 (~Telepressure * ~Techno-overload) demonstrates that simultaneously alleviating the expectation of immediate responsiveness and reducing the overall volume of digital tasks constitutes a highly consistent configuration associated with non-high burnout, irrespective of academic pressures. Strictly interpreted within the measured variables of the JD-R model, these asymmetrical pathways indicate that student-athletes can maintain psychological equilibrium as long as specific, overwhelming combinations of dual-role demands are structurally avoided (Stambulova et al., 2021).

#### 4.1.3. Synthesis of the Multi-Analytical Approach

Rather than employing a classical qualitative-quantitative mixed-methods design, this study utilized a dual-method quantitative approach to achieve deeper methodological integration. While PLS-SEM assessed the structural net effects indicating the indirect role of digital strain, it may mask the existence of multiple concurrent pathways. By disaggregating the composite “digital strain” construct into its specific dimensional components (telepressure, overload, conflict), the fsQCA revealed how distinct facets of digital demands interact synergistically with academic stress.

Nevertheless, the integration of these findings must be interpreted with extreme methodological caution. Reviewing the contrast between the methods, it is plausible that a variable demonstrating non-significant net effects in PLS-SEM might emerge as a component of a specific fsQCA configuration. Given the restricted sample size and the high internal correlation among digital strain dimensions, the specific configurational patterns identified via fsQCA might capture localized, subgroup-specific co-occurrences rather than universally robust phenomena. Therefore, while the PLS-SEM findings confirm the broad structural relevance of digital strain, the precise configurational combinations should be viewed as localized propositions mapping the complex reality of female student-athletes.

### 4.2. Theoretical Implications

This study provides specific theoretical contributions to the literature on student-athlete well-being by contextualizing the Job Demands–Resources (JD-R) model and the Stressor–Strain–Outcome (SSO) framework within the digitalized collegiate sports environment.

First, addressing the conceptualization of academic stress within the JD-R model, this study provides a theoretical rationale for treating academic obligations as a mandatory “job demand” for student-athletes. Unlike the general student population, collegiate athletes face rigid institutional requirements to maintain specific academic standards to preserve their athletic eligibility and scholarship status (Sorkkila et al., 2017). This institutional mandate endows academic stress with a sustained, resource-depleting nature that is functionally equivalent to occupational demands in traditional organizational settings. By modeling this structurally distinct stressor, the current research broadens the applicability of the JD-R model to the dual-career context.

Second, the study addresses the complexity of digital communication by adopting a focused conceptualization of digital strain. While digital platforms inherently possess the potential to function as informational resources—such as facilitating team coordination and distributing tactical knowledge—this study specifically isolates their maladaptive “dark side” (Tarafdar et al., 2020). By operationalizing digital strain as the amalgamation of telepressure (the internal urge to respond) and technostress (overload and conflict), the findings clarify that it is not the digital medium itself, but rather the boundary-blurring expectation of constant availability, that acts as a depleting demand.

Third, the research deepens the theoretical understanding of the student-athlete dual identity by highlighting how digital communication exacerbates inherent time conflicts. The roles of “student” and “athlete” continuously compete for limited temporal and cognitive resources (Stambulova et al., 2021). The findings suggest that when structural academic demands establish a baseline of resource depletion, the unpredictable, “always-on” nature of digital team management acts as a proximal catalyst for burnout. This demonstrates that in modern collegiate sports, role conflict is no longer confined to physical schedules but has extended into the digital space, bridging the gap between academic pressure and athletic exhaustion.

### 4.3. Practical Implications

Given the methodological constraints of this study—specifically the cross-sectional design, reliance on self-reported data, and a restricted sample of 67 female collegiate basketball players—the following practical implications are not presented as definitive interventions. Instead, they serve as potential exploratory directions for athletic departments that require rigorous testing through future intervention studies or short-term longitudinal tracking (e.g., utilizing three-month intervals across a competitive season).

From an organizational management perspective, athletic departments and coaching staff might consider evaluating the boundaries of digital team communication. Since prolonged telepressure and techno-overload operate as proximal conduits for emotional exhaustion, exploring the feasibility of establishing “digital quiet hours” could be a valuable strategy. For instance, limiting non-urgent tactical instructions or administrative notifications during late evening recovery periods might help mitigate techno-conflict without compromising team performance. Such exploratory measures aim to protect the essential recovery windows of student-athletes.

Furthermore, acknowledging the unique vulnerability generated by the convergence of high academic stress and digital overload, enhanced structural coordination between athletic coaching staff and academic advising units is recommended. During periods of peak academic demands, such as mid-term or final examination weeks, coaching staff might consider temporarily reducing the volume of digital tactical assignments. Proactively managing the intensity and timing of digital expectations may offer sports administrators a proactive approach to safeguarding athlete well-being within highly demanding dual-career environments.

## 5. Conclusions

This section synthesizes the core empirical insights derived from the research. It outlines the primary conclusions established by the dual-method analysis and systematically acknowledges the methodological limitations, providing specific and actionable directions for future investigations.

### 5.1. Summary of Findings

This multi-analytical study provides nuanced insights into the mechanisms through which dual-role demands are associated with athlete burnout in a digitalized collegiate sports environment. By employing variance-based PLS-SEM, the research identifies digital strain—specifically the combination of telepressure and techno-overload/conflict—as a critical proximal conduit linking structural academic stress to psychological exhaustion. The structural model indicates that academic demands deplete foundational resources, leaving student-athletes highly vulnerable to the acute, boundary-blurring nature of digital team communications. Furthermore, the set-theoretic fsQCA reveals that burnout is not triggered by a single isolated stressor, but rather emerges from specific configurations of dual-role demands. High academic stress combined with techno-overload, or a purely “digital saturation” state (high telepressure, overload, and conflict), constitute configurations sufficiently associated with high burnout within this calibrated sample. Conversely, strategically mitigating the expectation of immediate responsiveness (telepressure) and reducing the sheer volume of digital tasks (techno-overload) serve as exploratory configurations associated with non-high burnout. Ultimately, this study highlights that managing the digital boundaries of collegiate athletes is as critical as managing their physical training loads.

### 5.2. Limitations and Future Research Directions

Despite the methodological rigor applied, this study contains several inherent limitations that must be acknowledged when interpreting the findings.

First, the most prominent limitation is the restricted sample size and homogeneous composition (N = 67 female collegiate basketball players from a specific competitive division). As highlighted by the methodological constraints of structural equation modeling and configurational analysis, this small sample limits the statistical stability of the estimates and the generalizability of the findings. The specific configurations identified via fsQCA rely on data-driven percentiles specific to this cohort and may capture localized co-occurrences rather than universal thresholds. Future research must prioritize validating these theoretical models and configurational pathways using substantially larger and more diverse samples, encompassing male athletes, different sports disciplines (e.g., individual sports), and varying competitive levels.

Second, the study utilizes a cross-sectional design, which inherently precludes the establishment of definitive causal relationships. While the directional expectations were grounded in the Job Demands–Resources model, the current data can only confirm robust associational patterns and co-occurrences. To rigorously test the causal trajectories of digital strain and burnout, future studies should employ short-term longitudinal tracking designs. Given the rapid fluctuations in academic and athletic schedules, tracking athletes at shorter intervals—such as every three months across a competitive season—would provide a more accurate and dynamic understanding of how these dual-role stressors accumulate and interact over time.

Third, all variables were assessed using self-reported measures. While Harman’s single-factor test was conducted to preliminarily assess the presence of common method bias, we acknowledge that this statistical technique offers only a limited diagnostic scope and cannot definitively rule out the influence of shared method variance. Consequently, common method bias remains a potential residual limitation of this study. To enhance the robustness of future inquiries, researchers should prioritize the integration of longitudinal data collection, marker variable techniques, or the incorporation of objective behavioral metrics—such as screen-time logs or physiological markers—to better isolate the effects of digital strain from subjective self-reporting.

## Figures and Tables

**Table 1:** Demographic characteristics distribution of the CUBAL female basketball player sample (N = 67).

Variables	Category	f	%
Grade	Fre.	21	31.3
	Sop.	17	25.4
	Jun.	12	17.9
	Sen.	11	16.4
	Gra.	6	9
Level	No	59	88.1
	Grade 2 and above	8	11.9
Years	Short (0–3 years)	40	59.7
	Middle (3.1–6 years)	20	29.9
	Long (>6.1 years)	7	10.4
Time	Almost 0 min	6	9
	<30 min	32	47.8
	30–60 min	16	23.9
	60–120 min	6	9
	>120 min	7	10.4

Note: N = 67. Grade = Academic grade (Fre. = Freshman, Sop. = Sophomore, Jun. = Junior, Sen. = Senior, Gra. = Graduate); Level = Athletic classification (No = Unclassified, Grade 2 and above = National second-grade and above); Years = Playing tenure (Short = 0–3 years, Middle = 3.1–6 years, Long = >6.1 years); Time = Daily time spent handling online team notifications.

**Table 2:** Descriptive statistics and correlation coefficient matrix of core variables.

Variables	Mean	SD	1	2	3	4	5
1. Training Tenure	3.33	2.55	1				
2. Time	2.64	1.11	0.065	1			
3. AS	3.15	0.94	−0.108	0.118	1		
4. DS	3.24	0.87	−0.182	0.021	0.697 **	1	
5. AB	2.42	0.67	−0.255 *	−0.23	0.393 **	0.472 **	1

Note: SD = Standard Deviation. * *p* < 0.05, ** *p* < 0.01. AB = Athlete burnout; AS = Academic stress; DS = Digital strain. Time = Daily time spent handling online team notifications.

**Table 3:** Reliability and convergent validity assessment of first-order and second-order measurement models.

Constructs & Dimensions	1st-Order Loadings	2nd-Order Loadings	Cronbach’s α	CR	AVE
AB	-	-	0.831	0.897	0.746
Reduced Accomplishment	0.661~0.911	0.733	0.774	0.843	0.646
Devaluation	0.792~0.871	0.901	0.829	0.886	0.661
Emotional Exhaustion	0.790~0.878	0.942	0.889	0.919	0.693
AS	0.794~0.875	-	0.871	0.911	0.719
DS	-	-	0.908	0.942	0.844
Telepressure	0.775~0.880	0.906	0.88	0.911	0.671
Techno-conflict	0.766~0.882	0.92	0.795	0.879	0.708
Techno-overload	0.737~0.901	0.929	0.864	0.907	0.710

Note: AB = Athlete burnout (Devaluation, Emotional Exhaustion, and RSOA are the three dimensions, where RSOA stands for reduced sense of accomplishment); AS = Academic stress; DS = Digital strain (comprising Telepressure, Techno-conflict, and Techno-overload). AS is a first-order unidimensional construct; in the disjoint two-stage approach, its latent variable scores serve directly as second-order indicators, hence the second-order loading is computed as 1.000. CR = Composite reliability; AVE = Average variance extracted. “-” indicates that the construct has no corresponding higher-order or lower-order dimensional division.

**Table 4:** Discriminant validity (HTMT) matrix of the final structural model.

Latent Variables	1. AS	2. AB	3. DS
1. AS			
2. AB	0.44		
3. DS	0.793	0.525	

Note: HTMT = Heterotrait–Monotrait Ratio. AB = Athlete burnout; DS = Digital strain; AS = Academic stress.

**Table 5:** Results of the base structural model (without controls).

Path/Relationship	β	[95% CI]	t-Value	*p*-Value
Direct Effects				
AS → DS (H2)	0.724 ***	[0.600, 0.832]	12.408	<0.001
DS → AB (H3)	0.391 *	[0.088, 0.687]	2.575	0.010
AS → AB (H1)	0.127	[−0.222, 0.444]	0.732	0.464
Mediation (Indirect Effect)				
AS → DS → AB (H4)	0.283 *	[0.066, 0.510]	2.512	0.012
Model Fit (R^2^)				
Digital Strain	0.524		-	-
Athlete Burnout	0.241		-	-

Note: AB = Athlete burnout; AS = Academic stress; DS = Digital strain. β = Standardized path coefficient; CI = Percentile 95% confidence interval (based on 5000 bootstrapping resamples). * *p* < 0.05, *** *p* < 0.001.

**Table 6:** Results of the controlled structural model.

Path/Relationship	β	[95% CI]	t-Value	*p*-Value
Direct Effects				
AS → DS (H2)	0.706 ***	[0.556, 0.835]	9.965	<0.001
DS → AB (H3)	0.347 *	[0.013, 0.668]	2.104	0.035
AS → AB (H1)	0.164	[−0.129, 0.484]	1.048	0.295
Mediation (Indirect Effect)				
AS → DS → AB (H4)	0.245 *	[0.01, 0.473]	2.102	0.036
Control Variables → DS				
Time → DS	−0.049	[−0.206, 0.132]	0.563	0.573
Tenure → DS	−0.142	[−0.365, 0.067]	1.308	0.191
Grade → DS	0.115	[−0.066, 0.294]	1.249	0.212
Control Variables → AB				
Time → AB	−0.243 *	[−0.495, −0.016]	1.971	0.049
Tenure → AB	−0.139	[−0.434, 0.156]	0.895	0.371
Grade → AB	−0.120	[−0.296, 0.068]	1.284	0.199
R^2^				
Digital Strain	0.550		-	-
Athlete Burnout	0.342		-	-

Note: AB = Athlete burnout; AS = Academic stress; DS = Digital strain. β = Standardized path coefficient; CI = Percentile 95% confidence interval (based on 5000 bootstrapping resamples); f^2^ = Effect size. * *p* < 0.05, *** *p* < 0.001.

**Table 7:** Configurational analysis results for high athlete burnout and non-high athlete burnout.

Conditions	AB		~AB	
	H1	H2	L1	L2
Academic Stress (AS)	●			⊗
Telepressure		●		⊗
Techno-overload	●	●	⊗	
Techno-conflict		●		⊗
Parameters				
Consistency	0.824	0.855	0.799	0.859
Raw Coverage	0.686	0.667	0.783	0.607
Unique Coverage	0.09	0.071	0.201	0.025
Cases (N)	15	14	17	13
Solution Consistency		0.822		0.792
Solution Coverage		0.757		0.808

Note: ● = Condition present; ⊗ = Condition absent. Blank spaces indicate a “don’t care” condition. The table reports the intermediate solutions. Case numbers represent the sum of empirical instances within the logical truth table rows encompassed by each configuration. AS = Academic stress; AB = High athlete burnout; ~AB = Non-high athlete burnout.

## Data Availability

The raw data supporting the conclusions of this article will be made available by the authors, without undue reservation.

## Supplementary Materials

The following supporting information can be downloaded at: https://www.mdpi.com/article/10.3390/bs16101889/s1.

## Abbreviations

CUBAL	Chinese University Basketball Association League
PLS-SEM	Partial Least Squares Structural Equation Modeling
fsQCA	Fuzzy-set Qualitative Comparative Analysis
AB	Athlete Burnout
AS	Academic Stress
DS	Digital Strain
TEL	Telepressure
TS	Technostress
RSOA	Reduced Sense of Accomplishment
JD-R	Job Demands–Resources
PAS	Perception of Academic Stress
SSO	Stressor–Strain–Outcome
ABQ	Athlete Burnout Questionnaire
Fre.	Freshman
Sop.	Sophomore
Jun.	Junior
Sen.	Senior
Gra.	Graduate
CMB	Common Method Bias
AVE	Average Variance Extracted
HTMT	Heterotrait–Monotrait Ratio
VIF	Variance Inflation Factor

## Appendix A

**Table A1:** Full Truth Table for fsQCA.

fzas	fzdstele	fzdsoverload	fzdsconflict	Number	fzab	Raw Consist.	PRI Consist.	SYM Consist.
1	1	1	1	12	1	0.862418	0.752177	0.76428
0	1	1	1	2	1	0.859776	0.612022	0.612022
1	0	1	1	2	1	0.846205	0.59331	0.59331
1	0	1	0	1	1	0.843019	0.514019	0.514019
1	0	0	1	2	0	0.798519	0.346154	0.346154
1	1	0	1	1	0	0.796165	0.391949	0.391949
0	0	1	0	1	0	0.791604	0.272251	0.272251
1	0	0	0	1	0	0.779555	0.310869	0.310869
0	1	0	0	1	0	0.74132	0.226069	0.226069
0	0	0	1	1	0	0.722883	0.171548	0.171548
0	0	0	0	12	0	0.598944	0.216495	0.223602

Note: 1 = condition present; 0 = condition absent. fzas = Academic stress; fzdstele = Telepressure; fzdsoverload = Techno-overload; fzdsconflict = Techno-conflict; fzab = Athlete burnout (Outcome). ‘number’ indicates the frequency of empirical cases observed within each configuration. ‘raw consist.’ refers to the raw consistency score, while ‘PRI consist.’ denotes the proportional reduction in inconsistency, utilized to prevent simultaneous subset relations of both the outcome and its negation.
