# Supplementary material for: Academic Stress, Digital Strain, and Athlete Burnout Among Female Collegiate Basketball Players: A PLS-SEM and fsQCA Study
Source: Behav Sci (Basel). 2026 Oct 13;16(10):1889. doi: 10.3390/bs16101889 (PMC13701667; doi:10.3390/bs16101889)
Supplement: Supplementary file 1 [file behavsci-16-01889-s001.zip › behavsci-4384363-supplementary.pdf]

number	tel1	as1	tel2	ts2	as2	tel3	ts3	as3	
1	5	3	4	3	3	4	3	3	
2	5	5	5	5	4	5	5	5	
3	4	4	3	2	3	3	3	3	
4	5	5	3	2	2	2	2	5	
5	5	2	1	1	1	1	1	1	
6	5	4	3	3	4	4	3	5	
7	4	4	4	4	4	4	5	4	
8	4	4	3	3	3	4	3	3	
9	4	5	4	2	4	4	4	4	
10	5	4	5	4	3	5	5	4	
11	5	5	5	5	5	5	5	5	
12	3	4	2	2	2	2	2	2	
13	5	5	1	3	4	1	2	2	
14	5	5	3	4	5	4	4	5	
15	4	3	3	2	2	4	2	2	
16	5	2	1	1	1	1	1	1	
17	4	4	3	3	4	3	4	3	
18	3	4	2	3	3	3	3	3	
19	5	4	2	2	4	4	3	3	
20	4	3	4	3	3	4	4	4	
21	5	5	5	5	5	5	5	3	
22	5	3	3	3	4	4	4	4	
23	5	4	2	2	2	4	4	2	
24	4	3	2	3	2	4	2	2	
25	5	3	3	3	2	3	4	3	
26	5	4	5	2	3	1	3	3	
27	5	4	3	3	3	4	4	4	
28	5	4	2	2	4	3	3	4	
29	5	5	5	5	5	4	4	5	
30	5	3	3	3	3	3	3	3	
31	4	4	4	4	4	4	4	4	
32	4	4	3	3	3	4	3	3	
33	5	3	3	2	2	3	3	3	
34	2	4	2	1	1	1	1	4	
35	4	3	3	2	2	2	4	2	
36	5	4	4	3	3	4	4	3	
37	5	2	4	2	2	3	1	2	
38	5	4	3	4	3	5	4	3	
39	5	1	3	2	1	3	3	3	
40	5	1	1	1	1	3	1	1	
41	5	1	3	1	1	3	2	3	
42	4	1	3	3	1	3	2	1	
43	3	4	3	3	3	3	4	3	
44	5	3	3	3	3	4	3	2	
45	5	3	3	3	3	3	3	3	
46	4	3	2	3	2	4	3	2	
47	5	4	2	2	1	4	4	1	
48	4	4	4	4	4	4	4	5	
49	5	4	3	3	4	5	3	4	

50	4	3	3	3	3	4	3	4
51	5	3	2	1	3	4	2	2
52	5	4	5	2	4	5	4	4
53	2	4	1	1	3	1	1	1
54	3	3	3	3	3	3	3	3
55	5	3	3	4	3	3	3	4
56	5	3	1	1	1	2	4	4
57	5	3	2	3	2	3	3	4
58	5	5	3	3	5	3	3	5
59	5	4	3	3	5	4	4	4
60	4	4	2	2	4	3	3	2
61	5	3	1	1	3	4	1	2
62	5	4	1	1	3	1	1	3
63	5	4	5	3	5	3	3	3
64	5	3	4	1	3	5	5	3
65	4	2	2	2	2	2	2	2
66	4	2	1	1	1	2	1	1
67	5	4	3	3	4	4	2	4

To ensure the accuracy of the data analysis, all reverse-scored items from the original scales were rec

AB = Athlete burnout; AS = Academic stress; TEL = Telepressure; CR = Coach-athlete relationship; TS = Technost

tel4	ts4	as4	tel5	ts5	ts6	tel6	ts7	ts8
4	4	4	4	4	4	4	4	3
5	5	5	5	5	5	5	5	5
3	3	3	3	3	2	3	1	1
4	4	4	4	2	4	3	2	2
1	1	1	1	1	3	5	1	1
5	4	4	5	5	5	5	4	3
4	4	4	4	4	4	4	4	2
4	4	3	4	3	3	2	4	3
4	4	4	4	4	4	4	4	4
5	5	3	5	2	5	5	5	4
5	5	5	5	5	5	5	5	5
3	3	2	2	4	3	2	2	2
3	2	4	2	2	2	1	1	1
5	4	4	5	4	4	3	3	2
4	4	2	4	2	4	4	3	2
1	2	1	2	1	1	1	1	1
3	3	3	3	3	3	3	3	3
3	3	3	3	2	5	1	2	2
4	5	4	4	3	3	4	3	3
4	4	3	4	5	5	5	5	5
5	3	3	5	3	5	5	5	5
4	3	4	3	4	4	3	4	3
5	4	3	4	2	4	4	2	3
4	4	2	4	2	2	4	2	2
4	3	2	3	2	3	3	2	2
4	3	3	3	3	4	4	3	3
4	3	4	4	4	4	4	4	2
5	2	2	4	2	4	4	2	2
5	4	4	4	5	5	5	4	5
3	3	3	3	3	3	3	3	3
4	4	5	3	3	1	3	2	3
4	4	4	4	3	4	4	3	3
5	5	3	4	3	3	3	4	3
4	4	2	4	2	2	2	2	1
4	4	2	4	1	4	4	2	1
4	4	3	5	3	4	4	4	3
4	4	2	5	2	2	5	2	1
5	5	3	4	4	3	5	5	3
3	3	3	5	3	5	5	4	3
3	1	1	3	1	4	3	1	1
3	3	3	3	3	4	3	3	1
3	3	1	4	1	2	4	1	2
3	4	3	3	2	3	3	4	3
3	4	3	3	3	4	3	3	3
3	3	3	5	3	3	3	3	1
4	3	3	4	3	4	3	3	3
5	5	2	4	2	4	4	2	3
4	4	4	4	4	3	4	4	3
5	5	3	5	4	5	5	5	3

4	4	3	4	4	4	4	4	3
4	2	2	4	2	4	4	2	2
5	5	4	5	5	5	5	5	4
1	1	1	1	3	2	2	1	1
3	3	3	3	3	3	3	3	3
4	5	4	4	3	4	3	5	3
3	4	5	5	1	3	4	5	3
5	5	3	5	4	4	4	5	3
5	5	3	5	3	3	3	5	3
5	5	4	5	4	5	4	5	4
4	4	3	3	3	3	3	3	2
4	2	2	4	1	4	4	2	1
2	1	3	5	1	1	3	2	1
5	5	5	5	1	4	5	4	2
5	2	3	5	3	5	5	4	1
2	3	2	4	2	3	3	2	1
1	3	2	1	1	1	1	1	1
4	3	3	4	3	4	4	4	4

oded prior to statistical protelssing to ensure that higher values consistently represent higher levels of ress.

cr1	cr2	cr3	cr4	cr5	cr6	cr7	cr8	cr9
6	6	6	6	6	6	6	6	6
6	6	7	7	7	7	7	7	6
6	6	6	6	6	6	6	6	6
4	5	7	7	7	7	6	6	6
7	7	7	7	7	7	7	7	7
4	6	7	6	7	7	7	7	7
7	6	6	7	7	7	7	7	7
5	5	6	6	6	7	7	5	4
5	5	5	5	5	5	5	5	5
4	5	7	5	6	7	7	6	5
7	7	7	7	7	7	7	7	7
4	4	4	5	5	5	5	5	5
4	6	7	7	7	7	7	7	6
6	7	7	6	7	7	6	7	6
6	6	6	6	6	6	6	6	6
7	6	7	7	7	7	7	7	7
6	6	6	7	7	7	7	6	6
7	7	7	7	7	7	7	7	7
4	4	5	6	6	6	6	5	4
6	6	7	6	7	7	7	7	7
7	7	7	7	7	7	7	7	7
5	4	5	4	5	6	6	5	6
6	7	7	7	7	7	6	7	6
4	4	5	4	5	6	6	4	4
4	4	7	7	7	7	7	7	5
4	6	7	7	7	7	7	7	4
4	5	5	5	6	6	6	5	5
5	6	6	7	7	7	7	6	5
4	6	7	7	7	7	7	7	7
6	6	6	6	6	6	6	5	5
4	6	5	7	7	7	7	7	5
4	6	6	6	6	6	6	6	5
4	4	6	7	7	7	7	6	6
4	6	6	6	7	7	6	6	5
4	7	7	7	7	7	7	7	7
5	5	7	6	7	7	7	5	5
5	6	7	7	7	7	7	7	7
5	4	7	7	7	7	7	7	7
7	7	5	7	7	7	7	5	5
4	6	6	7	7	7	7	7	6
6	6	6	6	6	6	6	5	6
6	7	7	4	5	6	4	6	6
4	5	4	7	7	7	7	6	3
3	5	7	7	7	7	4	6	4
5	4	7	7	7	7	4	7	7
2	2	4	4	4	7	4	4	5
7	7	7	7	7	7	7	7	7
3	5	4	4	6	6	6	5	5
1	4	4	4	6	6	6	2	1

4	5	5	5	6	6	5	5	5
6	7	7	7	7	7	7	7	6
6	6	7	6	6	7	7	7	7
2	7	7	7	7	7	7	7	7
4	4	4	4	4	5	4	4	4
4	4	5	6	6	6	6	5	5
3	5	7	6	6	6	7	6	5
4	6	7	7	7	7	7	7	7
4	4	7	7	7	7	7	4	4
6	6	6	6	6	7	7	5	5
4	6	6	6	6	6	6	6	6
6	6	6	7	7	7	7	6	6
6	6	7	7	7	7	7	7	7
6	6	7	7	7	7	7	7	5
4	6	6	5	6	7	7	5	7
4	7	7	7	6	7	7	7	5
4	7	7	6	6	6	7	6	6
6	6	5	7	7	7	7	5	7

the measured construct.

cr10	cr11	ab4	ab5	ab12	ab6	ab7	ab2	ab3
6	6	4	2	3	2	2	3	2
7	7	4	3	4	5	3	4	4
6	6	2	1	1	2	2	2	2
6	6	5	5	2	3	4	4	1
7	7	1	1	1	1	1	1	1
7	7	4	3	3	2	3	3	3
7	7	3	3	2	4	3	2	2
6	7	3	3	4	3	3	2	3
5	5	3	3	3	3	3	3	3
5	5	4	2	4	2	3	3	2
7	7	1	1	1	1	1	1	1
5	5	3	3	3	3	3	3	3
6	6	2	1	2	2	3	2	2
6	7	3	3	3	2	2	4	2
6	6	2	2	2	2	2	2	2
7	7	1	1	1	1	1	1	1
6	7	3	4	3	2	3	3	2
7	7	2	2	2	1	1	2	2
4	7	3	3	3	2	3	3	1
7	7	4	4	3	3	3	4	3
7	7	3	3	3	3	3	3	3
7	6	3	4	3	5	4	4	4
7	7	2	1	1	2	3	2	2
5	6	3	4	3	3	4	2	1
7	7	1	2	1	1	4	1	1
6	7	4	3	3	3	3	2	2
6	6	4	3	3	3	3	4	3
6	6	1	1	2	2	4	3	2
7	7	4	3	3	2	3	3	3
5	6	3	3	3	1	2	2	2
5	5	4	3	2	2	5	5	3
6	6	3	2	3	2	3	3	2
6	7	3	2	2	2	3	3	2
7	7	2	2	1	3	4	3	2
7	5	1	1	1	1	3	1	1
6	6	3	3	4	3	3	3	3
7	7	3	2	2	1	3	3	1
7	7	3	3	3	2	2	3	3
7	7	3	3	3	2	3	2	2
6	7	2	1	2	1	4	2	2
6	6	3	3	2	2	4	3	2
7	7	5	2	4	3	3	3	3
4	5	3	4	3	3	4	3	3
4	6	2	5	2	3	5	2	4
7	7	2	2	2	3	2	1	1
6	7	3	3	3	2	4	4	3
7	7	3	2	2	2	2	1	1
6	6	4	4	3	4	4	4	2
5	6	3	5	3	3	3	4	3

5	5	3	2	4	3	3	4	2
7	7	3	2	2	2	2	3	2
7	7	2	1	2	1	2	2	1
7	7	2	2	2	2	2	2	2
4	4	3	3	3	3	3	3	3
5	7	3	3	3	3	3	3	3
7	7	4	3	2	2	5	3	1
7	7	3	2	2	2	3	2	1
7	7	3	1	2	3	3	3	2
5	7	3	3	4	3	3	4	2
6	6	2	3	2	3	3	2	1
6	6	2	2	2	3	2	2	2
7	7	3	1	1	1	2	1	2
7	7	3	3	3	3	3	4	3
4	6	3	3	3	5	4	3	3
7	7	1	1	1	1	4	1	1
5	6	2	2	2	3	3	2	1
7	7	3	2	3	2	4	3	2

ab8	ab9	ab13	ab10	ab15	ab14
4	2	2	2	2	2
5	4	3	4	2	3
2	1	1	1	1	2
1	1	4	1	1	3
1	1	1	1	1	5
3	3	2	3	2	3
3	2	3	2	2	3
3	3	3	3	3	3
3	3	3	3	3	3
3	2	3	3	2	3
1	1	1	1	1	1
3	3	3	3	3	3
2	2	2	2	2	3
2	3	2	2	1	4
2	2	2	2	2	2
1	1	1	1	1	3
3	2	4	2	2	4
2	1	2	1	1	3
3	2	2	3	2	3
4	3	3	3	2	3
3	2	3	3	3	3
5	4	4	4	4	3
2	2	4	2	2	4
1	3	4	2	2	4
4	1	3	1	1	5
2	2	3	2	2	4
4	2	3	3	2	4
3	2	3	2	1	3
3	2	3	3	2	3
2	2	2	2	2	4
3	2	5	2	2	5
3	2	3	3	2	4
3	1	3	3	3	4
1	1	2	1	1	1
1	1	3	1	1	4
4	3	3	3	2	3
3	1	3	1	1	5
3	1	2	2	2	3
2	1	3	2	1	3
2	2	2	2	1	2
1	1	1	1	1	4
5	3	4	3	3	4
3	3	4	3	2	4
3	1	5	1	1	4
1	1	1	1	1	3
2	2	3	2	1	5
1	1	1	1	1	2
4	3	3	3	2	3
3	1	4	3	1	4

2	3	4	2	3	5
2	2	2	2	2	3
1	1	1	1	1	5
2	2	2	2	2	3
3	3	3	3	3	3
3	3	3	3	2	4
2	1	3	1	1	3
3	2	3	3	1	4
3	2	3	2	2	3
3	2	5	3	2	4
2	1	3	1	1	4
2	1	3	2	2	3
2	1	1	1	1	2
4	2	3	3	1	4
2	2	5	3	3	5
1	2	4	1	1	4
2	2	3	2	1	4
3	2	2	3	4	3
